# Development and external validation of the HCH and HPMS prognostic indices for sepsis: a retrospective model development study using a Multi-Objective Non-Newtonian Fluid optimization algorithm

**DOI:** 10.1186/s12911-026-03609-8

**Published:** 2026-06-02

**Authors:** Chengcheng Gao, Haiyue Zhang, Rui Zhang, Nan Li, Yanfang Song, Ying Liang, Zhe Yang, Liang Li, Xiaowei Gao, Li Dong, Xun Jiang, Zhijun Tan, Lei Shang

**Affiliations:** 1 Department of Health Statistics, Ministry of Education Key Lab of Hazard Assessment and Control in Special Operational Environment, School of Public Health, Fourth Military Medical University, Xi’an, Shaanxi 710032 China; 2 Department of Nursing, Noncommissioned Officer School, Army Medical University, Shijiazhuang, Hebei 050081 China; 3 Department of Medical Quality Management, Xijing Hospital, Fourth Military Medical University, Xi’an, Shaanxi 710032 China; 4 94498 Military Hospital, Nanyang, Henan 473000 China; 5 Department of Pediatrics, Tangdu Hospital, Fourth Military Medical University, Xi’an, Shaanxi 710038 China

**Keywords:** Multi-objective optimization, Sepsis, Prognostic indicators, Prognosis prediction, Machine learning

## Abstract

**Background:**

The pathological heterogeneity of sepsis makes it challenging for traditional scoring systems to balance early-warning sensitivity, dynamic progression characterization, and mechanistic interpretability. Developing a multi-objective optimization algorithm incorporating rheological properties to screen novel composite parameters from routine indicators, thereby constructing a precise sepsis assessment tool with both clinical feasibility and pathological interpretability, offers a new approach to inform clinical decisions and prognosis prediction for sepsis patients. This retrospective prognostic model development and validation study aimed to develop and externally validate novel composite indices for sepsis mortality prediction.

**Methods:**

Using data from the eICU (derivation cohort, *n* = 3,965) and MIMIC-IV (temporal validation cohort, *n* = 2,917) databases, we developed the Multi-Objective Non-Newtonian Fluid Optimization (MONNF) algorithm through physically inspired dynamic viscoelastic regulation, multi-target synergistic constraints, and medical knowledge-embedding strategies integrated with key pathological dimensions of sepsis. This algorithm screened novel indicators capturing the pathophysiological interplay among circulatory compromise, coagulopathy, and metabolic derangement. Subsequently, an Under-sampling Synchronous Evolutionary Ensemble (USEE) prediction model was established to validate its cross-center generalizability and clinical utility.

**Results:**

The initial phase of MONNF identified the Hypoxia-Coagulation-Hemoglobin Index (HCH; components: lactate, INR, hemoglobin) with a 28-day mortality ROC-AUC of 0.67, superior to SOFA (ROC-AUC = 0.63). The later phase constructed the Hepatic-Pulmonary-Metabolic Synergistic Index (HPMS; components: total bilirubin, PaO₂/FiO₂, bicarbonate, albumin) with a ROC-AUC of 0.64. Risk inflection points of the new indicators revealed critical intervention windows (pnonlinear < 0.001), while Boruta feature importance ranking confirmed their higher decision weight than traditional variables. The USEE model demonstrated optimal predictive performance in independent validation (ROC-AUC = 0.84, PR-AUC = 0.71).

**Conclusion:**

In this retrospective development and validation study, a novel optimization algorithm based on dynamic non-Newtonian fluid properties successfully uncovered composite indicators that profoundly characterize core pathological features of sepsis. This work provides an innovative solution to simplify clinical stratification and address the tripartite paradox in prognostic assessment: efficiency versus sensitivity versus interpretability.

**Supplementary Information:**

The online version contains supplementary material available at 10.1186/s12911-026-03609-8.

## Highlights

Developed MONNF algorithm: A multi-objective model addressing sepsis pathological heterogeneity.

Identified HCH and HPMS indices: Capturing core pathological cascades in sepsis.

Enhanced early-warning: Evolutionary ensemble framework improving mortality risk prediction.

Ensured clinical interpretability: Feature importance aligned with pathological mechanisms.

**Supplementary Information:**

The online version contains supplementary material available at 10.1186/s12911-026-03609-8.

## Introduction

Sepsis is a dysregulated systemic inflammatory response syndrome triggered by infection, whose complex pathophysiological mechanisms involve a vicious cycle of metabolic dysregulation, coagulopathy, immune imbalance, and multi-organ dysfunction, resulting in approximately 11 million global deaths annually with a 28-day mortality rate of 20%-35% [1, 2]. As a multi-organ dysfunction syndrome caused by the host’s dysregulated response to infection, sepsis presents formidable challenges for prognosis assessment due to its heterogeneous pathophenotypes and dynamic progression. Although the latest Sepsis-3 international consensus recommends SOFA scoring for risk stratification [3], its clinical implementation still faces a threefold dilemma: (1) Alert sensitivity versus decision-making expediency: Traditional systems like SOFA require complex parameters from multiple organ systems (e.g., coagulation, hepatic/renal function), causing operational delays in emergency settings [4]; (2) Static assessment versus dynamic evolution: Most conventional scores rely on single time-point thresholds, failing to quantify the synergistic prognostic value of temporal metrics like lactate clearance or platelet trend monitoring for multi-organ failure prediction [5]; (3) Statistical correlation versus mechanistic interpretability: While novel machine learning models improve predictive accuracy, their computational opacity hinders targeted intervention in core pathological pathways. Developing novel prognostic instruments integrating clinical feasibility, dynamic sensitivity and pathological interpretability represents an urgent need in sepsis precision medicine [6].

Swarm Intelligence Optimization Algorithms (SIOA) offer a potential breakthrough for this challenge through multi-objective trade-offs (e.g., feature dimensionality versus model performance), enabling the extraction of streamlined indicator combinations from high-dimensional data providing informational support for clinical decision-making. However, existing studies reveal significant limitations in sepsis applications: (1) Algorithmic constraints: Classical algorithms (e.g., NSGA-II, MOPSO) are prone to local optima entrapment with high-dimensional medical data and lack self-adaptive search strategy modulation [7–9]; (2) Clinical-pathological disconnection: Most algorithms treat laboratory parameters as independent variables, neglecting the synergistic pathophysiological crosstalk central to sepsis, resulting in clinically uninterpretable indicator combinations [10–12]. Designing an autonomous optimization framework capable of sensing data distribution characteristics and integrating medical prior knowledge thus becomes critical for overcoming sepsis prognostic modeling challenges.

Resolving these challenges necessitates innovative algorithmic frameworks, and the distinctive rheological properties of non-Newtonian fluids offer biophysical insights: under external stress, their viscosity dynamically self-adjusts (e.g., shear thinning reduces local flow resistance, while die swell enhances spatial expansion). This “environment-behavior” adaptive mechanism profoundly aligns with the core requirements for sepsis prognostic indicator screening—algorithms must dynamically balance “global exploration” (discovering novel cross-organ interaction patterns) and “local exploitation” (capturing precise critical thresholds) within complex data topologies [9, 13]. Based on this, we propose a Multi-objective Non-Newtonian Fluid Optimization (MONNF) algorithm. Through bidirectional iterative optimization integrating algorithmic and pathological logic, this method not only embeds clinically driven indicator processing strategies into the optimization process to enhance model interpretability and medical rationality, but also constructs through cross-system integration novel indicators that are more concise and sensitive than conventional scores.

## Data and method

### Study design and reporting guidelines

**Fig. 1:**
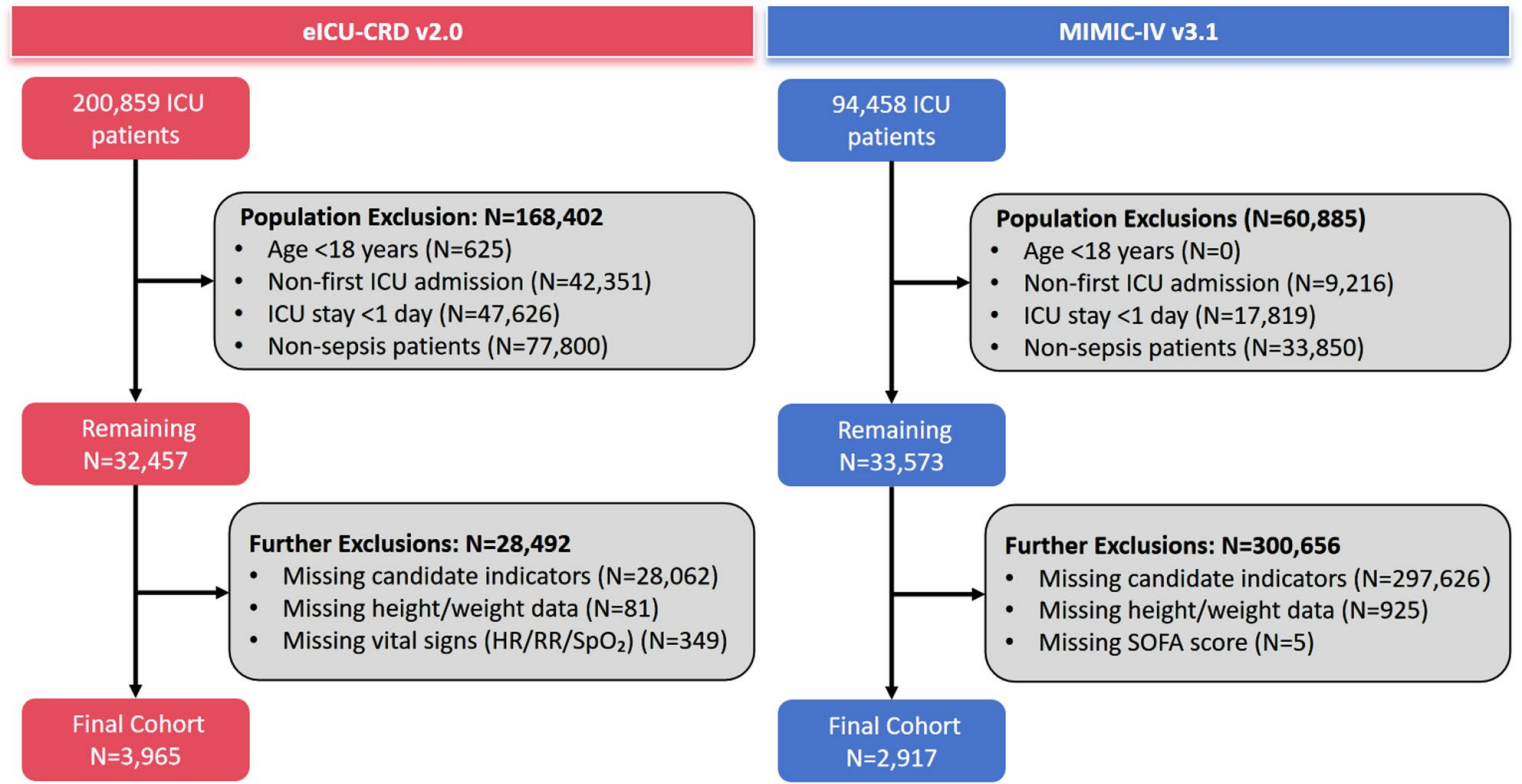
Subject enrollment workflow

This study is a retrospective, multi-center cohort study for the development and external validation of prognostic indices and a predictive model for sepsis. The study design involved: (1) using the eICU-CRD v2.0 database as the derivation cohort; (2) using the MIMIC-IV v3.1 database as a distinct, temporally separate external validation cohort to assess generalizability; and (3) pre-specifying the primary outcome as 28-day all-cause mortality (certification number: 12082112). We enrolled 6,882 adult patients with sepsis (eICU-CRD: 3,965 patients; MIMIC-IV: 2,917 patients), excluding those: (1) aged < 18 years, (2) with ICU stays < 24 h, or (3) lacking key baseline data (see Fig. 1 for screening workflow). The study protocol was not formally registered but followed a pre-defined analysis plan. This manuscript was prepared in strict accordance with the Transparent Reporting of a multivariable prediction model for Individual Prognosis Or Diagnosis–Artificial Intelligence (TRIPOD + AI) reporting guideline [14]. The completed TRIPOD + AI checklist is provided as Supplementary File 1.

**Fig. 2:**
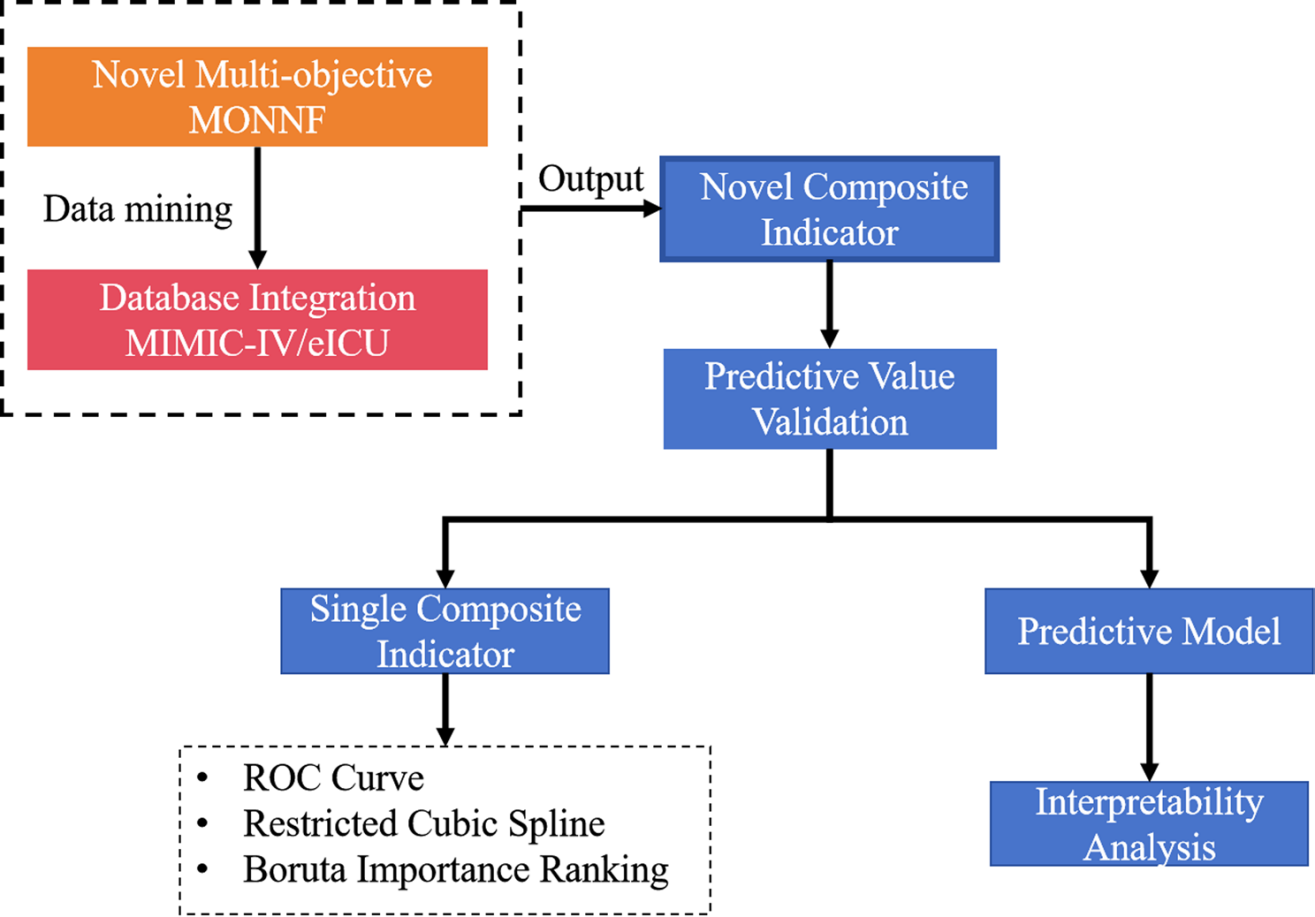
Technical workflow diagram

The research workflow comprises: first, mining the integrated MIMIC-IV/eICU database through the innovatively developed non-Newtonian fluid multi-objective optimization algorithm to extract novel composite indicators characterizing sepsis’ core pathological mechanisms; then systematically validating the predictive value of these indicators by: performing multi-dimensional assessment at the single-indicator level (including ROC curve discrimination efficacy analysis, restricted cubic spline nonlinear relationship identification, and Boruta feature importance ranking); constructing predictive models at the combinatorial application level; and ultimately conducting SHAP interpretability analysis based on model outputs to dissect indicator decision-contribution mechanisms. The technical roadmap is presented in Fig. 2.

### Variable extraction and outcome measures

The study analyzed clinically measured indicators obtained within the first 24 h of admission, specifically including: demographic indicators: age, sex, race, and ICU admission types; real-time physiological parameters: body mass index (BMI), heart rate (bpm), respiratory rate (times/min), peripheral oxygen saturation (SpO₂); comorbidity status: diabetes mellitus, congestive heart failure, chronic liver disease, chronic kidney disease, chronic obstructive pulmonary disease, cerebrovascular disease, peripheral vascular disease, malignant tumors, and acquired immunodeficiency syndrome (AIDS), with overall comorbidity burden quantified using the Charlson Comorbidity Index (CCI); disease severity scores: Simplified Acute Physiology Score II (SAPS II), Oxford Acute Disease Severity Score (OASIS), Sequential Organ Failure Assessment (SOFA); laboratory and metabolic indicators: serum lactate, blood glucose, white blood cell count (WBC), neutrophil-to-lymphocyte ratio (NLR), creatinine, total bilirubin, oxygenation index (PaO₂/FiO₂), bicarbonate, albumin, hemoglobin, platelet count, and international normalized ratio (INR).

The primary endpoint was 28-day all-cause mortality after ICU admission, with secondary endpoints including 90-day all-cause mortality and ICU length of stay (analyzed only for surviving patients). All variable definitions and collection time points strictly adhered to the Sepsis-3.0 diagnostic criteria.

Sepsis-3 Operational Definition: To ensure auditability, Sepsis-3 was implemented using following EHR logic: (1) Suspected Infection: Identified by the co-occurrence of antibiotic administration and microbiological cultures. Following clinical criteria, if a culture was obtained first, the antibiotic must be given within 72 h; if the antibiotic was given first, the culture must be ordered within 24 h. (2) Baseline SOFA: Assumed to be 0 for patients without pre-existing organ dysfunction. For patients with known chronic comorbidities, the baseline was estimated using the earliest available data before the infection window. (3)SOFA Timing: Sepsis was defined as an acute SOFA increase of ≥ 2 points within a window from 48 h before to 24 h after the suspected infection time, capturing the peak severity.

### Variable processing strategy

Based on the core pathological mechanisms of sepsis (including metabolic dysregulation, inflammatory imbalance, and organ damage), this study selected key prognostic indicators from the following six dimensions: (1) Metabolism and tissue hypoxia; (2) Inflammatory response intensity; (3) Organ dysfunction; (4) Oxygenation and acid-base balance; (5) Nutrition and repair; (6) Coagulation and endothelial injury.

**Fig. 3:**
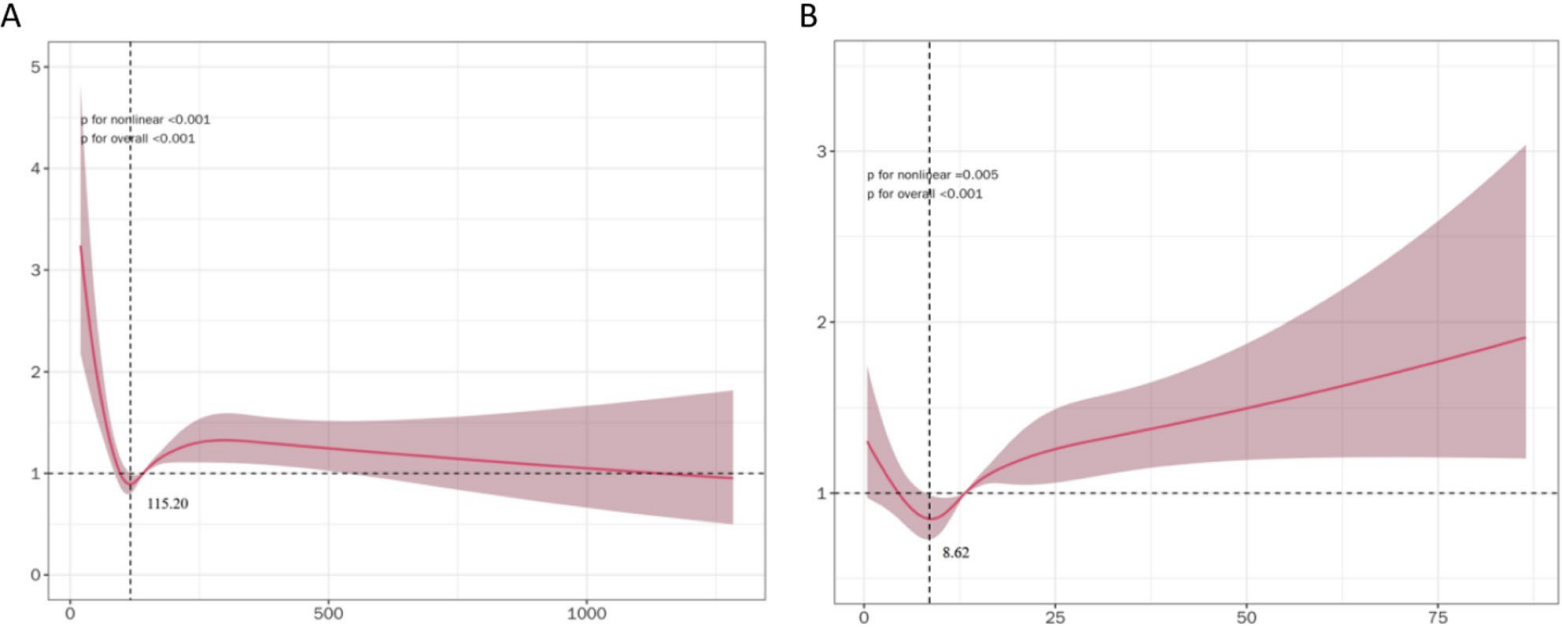
Nonlinear association analysis between blood glucose/white blood cell count and 28-day mortality risk. Note: Both analyses represent single-indicator logistic regression models using restricted cubic splines; **A**: Blood glucose (knot number = 5; inflection point = 115.20 mg/dL); **B**: White blood cell count (knot number = 5; inflection point = 8.62 × 109/L)

Differential mathematical processing strategies were developed based on three complementary evidences: (a) literature-reported pathophysiological mechanisms (references in Table 1 “Rationale for Inclusion”), (b) clinical consensus (Sepsis-3 guidelines), and (c) pre-analysis using restricted cubic splines (U-shaped associations of glucose/WBC confirmed in Sect.  3.1, Fig. 3).

To ensure the resulting indicators are intuitive and suitable for rapid bedside prognostic assessment (similar to the construction of the Advanced Lung Cancer Inflammation Index ALI or FIB-4), we converted all variables into a unified “high-value-high-risk” format before integrating them into the composite index:

1. Direct Risk Components (Positive correlation): For indicators where abnormal elevation directly reflects increased risk (e.g., lactate), original values were incorporated to represent the intensity of physiological stress.
2. Inverse Risk Components (Reciprocal transformation): For indicators where a numerical decrease indicates poorer outcomes (e.g., albumin), we applied a reciprocal transformation (1/x). This approach, placing protective factors in the denominator, is a standard heuristic in clinical index development, allowing the composite score to amplify the risk signal as these protective values decline [15, 16].
3. Homeostatic Deviation Components (U-shaped association): For variables exhibiting U-shaped risks (e.g., glucose, WBC), we quantified the risk as the absolute deviation from a defined physiological threshold. This method assumes that any departure from the homeostatic target—whether hyper- or hypo-state—contributes to physiological instability. This logic mirrors the “penalty points” system used in the APACHE II and Modified Early Warning Score (MEWS), where risk is assessed by the magnitude of deviation from normal physiological intervals [17].

All indicators utilized the first measured values within 24 h of admission to capture the potential impact of early laboratory findings on prognosis in sepsis. Detailed processing parameters are presented in Table 1.

**Table 1:** Candidate pool of prognostic indicators

Indicator Category	Association with Poor Prognosis	Processing Strategy	Rationale for Inclusion
**Metabolism and Tissue Hypoxia**			
Lactic acid (Lac)	High	Direct Risk Component (Value)	*Reflects tissue hypoperfusion/hypoxia*,* strongly associated with septic shock*,* MODS*,* and mortality *[18, 19]
Glucose (Glu)	High/Low	Homeostatic Deviation: |Glu-A|	*Both hyper-/hypoglycemia linked to immune dysregulation and endothelial injury; derived U-shaped threshold deviation *[20, 21]
**Inflammatory Response Intensity**			
White blood cell (WBC)	High/Low	Homeostatic Deviation: |WBC-B|	*Elevated WBC indicates hyperinflammation; reduced levels suggest immunosuppression (U-shaped threshold deviation*) [22, 23]
Neutrophil-to-lymphocyte ratio (NLR)	High	Direct Risk Component (Value)	*NLR = neutrophils/lymphocytes; quantifies systemic inflammation-immunosuppression imbalance; high NLR predicts worse outcome*s [24, 25]
**Organ Dysfunction**			
Creatinine (Cr)	High	Direct Risk Component (Value)	*Marker of renal impairment (AKI)*,* reflecting fluid management and metabolic waste clearance capacity *[26, 27]
Total bilirubin (TBIL)	High	Direct Risk Component (Value)	*Indicates hepatic injury/cholestasis*,* suggesting hepatobiliary involvement and impaired toxin clearance *[28, 29]
**Oxygenation and Acid-Base Balance**			
Oxygenation index (OI)	Low	Inverse Risk Component (1/Value)	*Gold standard for lung injury; low values predict ARDS risk (requires mechanical ventilation data) *[30]
Bicarbonate (HCO₃⁻)	Low	Inverse Risk Component (1/Value)	*Low HCO*₃^-^ *reflects hypoperfusion*,* metabolic/lactic acidosis *[31]
**Nutrition and Repair**			
Albumin (Alb)	Low	Inverse Risk Component (1/Value)	*Hypoalbuminemia indicates inflammatory consumption*,* capillary leakage*,* and worsening nutritional status *[19, 32]
Hemoglobin (Hb)	Low	Inverse Risk Component (1/Value)	*Anemia exacerbates oxygen delivery failure*,* correlating with energy metabolism and multi-organ support demands *[33, 34]
**Coagulation and Endothelial Injury**			
Platelets (PLT)	Low	Inverse Risk Component (1/Value)	*Sepsis-induced thrombocytopenia signals coagulopathy activation*,* DIC risk*,* and endothelial damage *[35, 36]
International normalized ratio (INR)	High	Direct Risk Component (Value)	*Elevated INR marks coagulation dysfunction*,* potentially progressing to DIC *[37]

### Novel indicator optimization

1. Algorithm Development

To address the limitations of traditional swarm intelligence algorithms (e.g., particle swarm optimization, ant colony optimization) in nonlinear, multimodal optimization scenarios, this study proposes a Non-Newtonian Fluid Optimization algorithm (NNF). The core innovation lies in abstracting evolutionary patterns from the rheological properties of non-Newtonian fluids (such as shear thinning, die swell, and tubeless siphoning) to construct dynamic adaptation mechanisms: during the initial exploration of the solution space, the algorithm mimics “shear thinning” to reduce viscosity for rapid global search; when approaching potential extremal regions, the “die swell” effect enhances local exploitation precision, while the “tubeless siphoning” characteristic enables cross-region gradient jumps to avoid local optima. Building upon this, a multi-objective extension strategy (Multi-Objective NNF, MONNF) is introduced, which hierarchically classifies population individuals through non-dominated sorting: first identifying non-dominated solutions to form the initial Pareto frontier, then iteratively calculating domination counts to progressively eliminate inferior solutions, ultimately dynamically maintaining the diversity and convergence of the Pareto frontier. Algorithm principles and simulation tests are detailed in the Supplementary File 2.

(2) Variable Allocation Mechanism

Within the multi-objective optimization framework of the MONNF algorithm, the assignment of variables to the numerator (N) or denominator (D) is autonomously optimized and determined by the algorithm. The specific logic is as follows: (a) Objective Function-Driven Process: The algorithm automatically evaluates how each variable’s allocation to the numerator/denominator position contributes to the model’s overall predictive performance by maximizing ROC-AUC (Objective 1) and minimizing the number of features/metrics used (Objective 2). (b) Clinical Plausibility Constraints: Directional consistency is ensured through predefined medical rules. (c) Population-Based Intelligent Decision-Making: During the Pareto optimal solution search, the algorithm evaluates tens of thousands of numerator/denominator combinations via non-dominated sorting. The final output preserves solutions demonstrating both clinical relevance and superior statistical performance.

(3) Optimization Modeling

With a known set of candidate indicators $M=\left\{ {{I_1},{I_2},...,{I_N}} \right\}$ associated with sepsis prognosis, the construction of a composite indicator *S* that balances predictive power and simplicity requires solving a multi-objective optimization problem: simultaneously maximizing its predictive performance for 28-day mortality outcomes while minimizing the number of included indicators. This is achieved by selecting partial indicators as numerator and denominator terms to construct a multiplicative ratio formula:

  

$$S=\frac{{{\prod _{j \in Numerator}}I_{j}^{{{w_j}}}}}{{{\prod _{k \in Denominator}}I_{k}^{{{w_k}}}}}$$

where *w*_*j*_ and *w*_*k*_ are binary decision variables (1 if the indicator is selected, 0 otherwise), with a minimum requirement of one indicator each for the numerator and denominator (i.e., $\sum {{w_j} \geqslant 1}$, $\sum {{w_k} \geqslant 1}$).

The formulated optimization problem involves two conflicting objectives. Primarily, Objective 1 seeks to maximize predictive efficacy, where the predictive capability of composite indicator S for 28-day mortality outcomes is evaluated through the area under the receiver operating characteristic curve (ROC-AUC):

  

$$\hbox{max} {\text{ }}{f_1}=AUC(S,Deat{h_{28d}})$$

Objective 2 aims to minimize the number of indicators, which reduces the incorporated indicators to enhance clinical feasibility, defined as the total count of selected items:

  

$$\hbox{min} {f_2}=\sum\limits_{{j=1}}^{N} {{w_j}} +\sum\limits_{{k=1}}^{N} {{w_k}} $$

### Predictive value analysis

1. Single-indicator Analysis

For the newly screened composite indicators, a multi-level validation strategy was employed to evaluate their independent predictive value: ROC curve analysis was used to assess the discriminative ability of individual indicators by calculating the area under the curve (AUC-ROC) and optimal cutoff values; restricted cubic splines (RCS) were applied to model the nonlinear dose-response relationship between indicators and adverse outcomes; the Boruta all-relevant feature importance ranking algorithm was integrated to quantify the global contribution of each indicator.

(2) Predictive Model Construction

An under-sampling synchronous evolutionary ensemble (USEE) model was developed based on the new indicators and baseline characteristics, which simultaneously optimized imbalanced data processing (under-sampling) and hyperparameter adaptive evolution (particle swarm optimization) to enhance model robustness [38]. For comparative purposes, conventional algorithms including LightGBM and XGBoost were implemented. All comparison models employed 5-fold cross-validation with grid search parameter tuning, and uniformly utilized SMOTE to address class imbalance. The eICU dataset served as the training set for model development, while the MIMIC dataset was used as an external test set to validate generalization capability, with evaluation conducted from both classification performance and clinical interpretability perspectives.

Classification Performance Evaluation: Precision (PRE), sensitivity (SEN), specificity (SPE), and accuracy (ACC) were computed to respectively reflect positive predictive precision, high-risk population identification ability, low-risk population exclusion capacity, and overall discriminative performance; F1-score was adopted to balance false-positive and false-negative risks, AUC-ROC was applied to assess comprehensive discriminative power, and AUC-PR was implemented to verify performance stability under imbalanced data conditions. Calibration curves were combined with Brier scores (lower values indicate more accurate predictions) to assess the accuracy of probabilistic predictions [39].

Clinical Interpretability Analysis: The SHAP (Shapley Additive Explanations) algorithm based on game theory was established to quantify feature contributions. This interpretable framework analyzed feature importance at both global and individual levels, measured each variable’s positive/negative weight on prognostic risk, elucidated association patterns between key biomarkers and clinical endpoints, and ultimately achieved visual interpretation of model prediction logic [40]. SHAP provides post-hoc feature attribution based on game theory, which quantifies predictive associations rather than biological causality. Results may be unstable under multicollinearity. Thus, SHAP outputs should be interpreted as hypothesis-generating signals for further mechanistic investigation [41].

### Statistical methods

For continuous variables, the Kolmogorov-Smirnov test was employed to assess normality. Normally distributed data were presented as mean ± standard deviation, with between-group comparisons analyzed using t-tests. Non-normally distributed data were expressed as median (interquartile range), with Mann-Whitney U tests applied for between-group difference analysis. Categorical variables were presented as percentages, with χ² tests used for between-group comparisons. Two-tailed tests were adopted for all significance tests, with P-value < 0.05 considered statistically significant. Data management was performed using PostgreSQL 16 for data extraction, while all statistical computations and model training processes were implemented in MATLAB R2024b. As a retrospective observational study, our findings represent associations rather than causal effects. Predictive models and indicator interpretations should be viewed through an associative lens, pending validation in interventional settings [42].

## Result

### Baseline characteristics of study population

The study included 6,882 sepsis patients, of whom 1,711 (24.9%) experienced 28-day mortality. Baseline characteristic analysis (Table 2) revealed that compared to the survival group, nonsurvivors were older in both cohorts (median age: eICU 70.0 vs. 65.0 years; MIMIC-IV 67.9 vs. 62.7 years, all *p* < 0.001). Among laboratory parameters, all except white blood cell count and glucose showed statistically significant between-group differences (all *p* < 0.05).

Restricted cubic spline modeling for non-linear association testing identified U-shaped dose-response relationships for both glucose (inflection point A: 115.20 mg/dL) and WBC count (inflection point B: 8.62 × 10⁹/L) (Fig. 3, all p-nonlinear < 0.001), demonstrating critical threshold effects on mortality risk.

**Table 2:** Baseline characteristics of study population

Characteristic	eICU-CRD v2.0			MIMIC-IV v3.1		
	Death (*n* = 849)	Survival (*n* = 3116)	*p*-value	Death (*n* = 862)	Survival (*n* = 2055)	*p*-value
**Basic characteristics**						
Age (years), median [IQR]	70.00 [60.0, 79.0]	65.00 [54.0, 76.0]	< 0.001	67.92 [56.9, 77.7]	62.72 [52.1, 72.8]	< 0.001
Gender, n(%)			0.679			0.920
Female	394 (46.41%)	1471 (47.21%)		344 (39.91%)	816 (39.71%)	
Male	455 (53.59%)	1645 (52.79%)		518 (60.09%)	1239 (60.29%)	
Race/Ethnicity, n(%)			0.920			< 0.001
Asian	11 (1.30%)	55 (1.77%)		20 (2.32%)	65 (3.16%)	
Black/African American	72 (8.48%)	276 (8.86%)		71 (8.24%)	192 (9.34%)	
Caucasian	674 (79.39%)	2427 (77.89%)		475 (55.10%)	1271 (61.85%)	
Hispanic/Latino	46 (5.42%)	178 (5.71%)		28 (3.25%)	82 (3.99%)	
Native American	8 (0.94%)	31 (0.99%)		1 (0.12%)	5 (0.24%)	
Other/Unknown	38 (4.48%)	149 (4.78%)		267 (30.97%)	440 (21.41%)	
Admission unit type, n (%)			0.431			< 0.001
Cardiac related Intensive Care Unit	145(17.08)	481(15.44)		185(21.46)	523(25.45)	
Medical intensive care unit (MICU)	627(73.85)	2304(73.94)		505(58.58)	943(45.89)	
Neuro-related intensive care unit	22(2.59)	99(3.18)		24(2.78)	46(2.24)	
Surgical intensive care unit (SICU)	55(6.48)	232(7.45)		148(17.17)	543(26.42)	
**Vital Signs**,** median [IQR]**						
BMI (kg/m^2^)	27.04 [22.8, 33.1]	28.29 [23.7, 34.7]	< 0.001	27.76 [24.1, 33.6]	28.71 [24.6, 33.7]	0.042
Heart Rate (bpm)	100.0 [82.0, 115.0]	98.0 [82.0, 113.0]	0.174	96.0 [82.0, 113.0]	93.0 [80.0, 109.0]	0.001
Respiratory Rate(breaths/min)	22.00 [18.0, 27.0]	21.00 [17.0, 26.0]	< 0.001	22.00 [18.0, 27.0]	20.00 [16.0, 25.0]	< 0.001
SpO₂ (%)	97.00 [93.0, 100.0]	98.00 [94.0, 100.0]	< 0.001	97.00 [93.0, 100.0]	98.00 [95.0, 100.0]	< 0.001
**Comorbidities**						
Diabetes, n(%)	250 (29.45%)	1073 (34.44%)	0.006	278 (32.25%)	658 (32.02%)	0.903
Congestive Heart Failure, n(%)	173 (20.38%)	603 (19.35%)	0.504	327 (37.94%)	699 (34.01%)	0.043
Chronic Liver Disease, n(%)	71 (8.36%)	146 (4.69%)	< 0.001	272 (31.55%)	540 (26.28%)	0.004
Chronic Kidney Disease, n(%)	165 (19.43%)	549 (17.62%)	0.222	241 (27.96%)	468 (22.77%)	0.003
Chronic Pulmonary Disease, n(%)	194 (22.85%)	652 (20.92%)	0.225	260 (30.16%)	528 (25.69%)	0.013
Cerebrovascular Disease, n(%)	94 (11.07%)	335 (10.75%)	0.790	148 (17.17%)	237 (11.53%)	< 0.001
Peripheral Vascular Disease, n(%)	56 (6.60%)	174 (5.58%)	0.263	103 (11.95%)	245 (11.92%)	0.984
Malignancy, n(%)	152 (17.90%)	364 (11.68%)	< 0.001	130 (15.08%)	247 (12.02%)	0.025
AIDS, n(%)	3 (0.35%)	9 (0.29%)	0.762	7 (0.81%)	23 (1.12%)	0.453
Charlson Comorbidity Index, median [IQR]	5.0 [3.0, 7.0]	4.0 [2.0, 6.0]	< 0.001	6.0 [4.0, 8.0]	5.0 [3.0, 7.0]	< 0.001
**Severity Scores**,** median [IQR]**						
SAPS II	58.0 [45.0, 69.0]	43.0 [33.0, 55.0]	< 0.001	56.0 [46.0, 68.0]	44.0 [36.0, 55.0]	< 0.001
OASIS	40.0 [33.0, 47.0]	34.0 [26.0, 41.0]	< 0.001	44.0 [37.8, 49.0]	38.0 [33.0, 44.0]	< 0.001
SOFA	4.0 [2.0, 6.0]	3.0 [2.0, 5.0]	< 0.001	3.0 [1.0, 6.0]	2.0 [0.0, 5.0]	< 0.001
**Laboratory Findings**,** median [IQR]**						
Lactate (mmol/L)	3.30 [1.8, 6.3]	2.20 [1.3, 3.8]	< 0.001	2.70 [1.6, 5.4]	1.80 [1.2, 2.9]	< 0.001
Glucose (mg/dL)	143.00 [107.0, 199.5]	142.00 [110.0, 199.0]	0.631	145.00 [108.0, 205.0]	137.00 [108.0, 181.0]	0.004
WBC (×10^9^/L)	14.00 [9.3, 20.0]	13.50 [9.2, 19.4]	0.212	14.25 [9.0, 20.8]	12.20 [8.0, 17.8]	< 0.001
NLR	11.71 [5.5, 22.4]	10.41 [5.4, 18.2]	0.018	13.01 [6.9, 23.4]	9.47 [5.2, 17.6]	< 0.001
Creatinine (mg/dL)	1.71 [1.1, 2.8]	1.40 [0.9, 2.4]	< 0.001	1.65 [1.0, 2.8]	1.20 [0.8, 1.9]	< 0.001
Total Bilirubin (mg/dL)	0.80 [0.5, 1.6]	0.60 [0.4, 1.1]	< 0.001	0.90 [0.4, 2.2]	0.80 [0.4, 1.7]	0.002
PaO₂/FiO₂ Ratio	173.70 [100.0, 290.5]	207.50 [120.0, 325.0]	< 0.001	192.50 [117.9, 294.0]	222.50 [136.0, 338.0]	< 0.001
Bicarbonate (mmol/L)	21.00 [17.0, 26.0]	23.00 [19.0, 27.0]	< 0.001	20.00 [16.0, 24.0]	22.00 [18.0, 25.0]	< 0.001
Albumin (g/dL)	2.60 [2.1, 3.1]	2.90 [2.4, 3.5]	< 0.001	2.80 [2.3, 3.3]	3.00 [2.5, 3.4]	< 0.001
Hemoglobin (g/dL)	11.10 [9.4, 12.9]	11.70 [9.8, 13.5]	< 0.001	10.40 [8.7, 12.1]	10.80 [9.1, 12.8]	< 0.001
Platelet Count (×10³/µL)	197.00 [127.0, 271.0]	223.00 [155.0, 298.0]	< 0.001	177.00 [106.8, 259.3]	188.00 [124.0, 266.0]	0.004
INR	1.50 [1.2, 2.1]	1.20 [1.1, 1.6]	< 0.001	1.50 [1.2, 2.2]	1.30 [1.2, 1.7]	< 0.001

### Prognostic indicator optimization

1. First-Round Screening

The multi-objective optimization screening strategy based on the MONNF framework achieved steady-state convergence after 1,000 iterations, yielding five non-dominated solution sets from the Pareto optimal frontier (Table 3), with feature dimensions ranging from 2 to 6 indicators. Comprehensive analysis of performance characteristics under multi-parameter optimization revealed stabilized AUC improvements beyond 3 indicators.

Adhering to the principle of indicator sparsity, the optimal combination was ultimately selected as an interactive triad comprising lactate, international normalized ratio (INR), and hemoglobin. This combination holistically represents the pathophysiological mechanisms of circulatory perfusion impairment and coagulation-oxygen transport decompensation, hence termed the Hypoxia-Coagulation-Hemoglobin Index (HCH Index). The computational formula is as follows:

  

$$HCH=\frac{{Lac \times INR}}{{Hb}}$$

**Table 3:** First-round optimization: non-dominated solution sets from pareto frontier

ID	Lac (*N*/D)	TBIL (*N*/D)	OI (*N*/D)	HCO₃⁻ (*N*/D)	Hb (*N*/D)	INR (*N*/D)	OBJ2 (Indicator Count)	OBJ1 (AUC)
1	N	—	—	—	—	N	2(2 + 0)	0.6629
2	N	—	—	—	D	N	3(2 + 1)	**0.6705**
3	N	—	D	D	—	N	4(2 + 2)	0.6739
4	N	N	D	D	—	N	5(3 + 2)	0.6811
5	N	N	D	D	D	N	6(3 + 3)	0.6846

Note: N: Feature included in numerator; D: Feature included in denominator; -: Feature not selected; OBJ 1: Bold indicates the selected optimal solution. OBJ 2: (Numerator count + Denominator count) in parentheses. Variable allocation is automatically optimized by the MONNF algorithm according to the following rules: Positive risk prognosticators are assigned exclusively to the numerator, negative protective indicators are restricted to the denominator, and U-shaped variables use |actual value - inflection point| in the numerator

(2) Second-round Screening

In the second phase of the hierarchical optimization framework, this study employed a complementary strategy by systematically excluding the three core indicators selected in the first screening round, shifting focus to conduct secondary mining of potential candidate indicators not originally included in the feature set. After 1,000 iterations, the algorithm converged to a stable state, yielding three non-dominated solution sets from the Pareto frontier (Table 4), with dimensional distributions of 2 to 4 composite parameter combinations.

Experimental results demonstrated that all candidate models exhibited discriminative performance below the predefined threshold (AUC < 0.65). Guided by the optimal compromise principle for weakly supervised scenarios, the final selection based on model efficacy prioritization yielded an optimal ratio structure: an interactive gradient scale integrating total bilirubin (TBIL), oxygenation index (OI), bicarbonate (HCO_3_⁻), and albumin (Alb). This composite deeply captures clinical phenotypes of hepatic metabolic dysfunction, oxygenation impairment, and nutritional imbalance, hence designated as the Hepatic-Pulmonary-Metabolic Synergistic Index (HPMS Index), with its formula presented below:

  

$$HPMS=\frac{{{\mathrm{TBIL}}}}{{{\mathrm{OI}} \times HCO_{3}^{ - } \times Alb}}$$

**Table 4:** Second-stage optimization: pareto frontier non-dominated solution sets

ID	TBIL (*N*/D)	OI (*N*/D)	HCO3⁻ (*N*/D)	Alb (*N*/D)	OBJ2 (Indicator Count)	OBJ1 (AUC)
1	N	—	—	D	2(1 + 1)	0.6271
2	N	D	—	D	3(1 + 2)	0.6364
3	N	D	D	D	4(1 + 3)	**0.6438**

Note: N: Feature included in numerator; D: Feature included in denominator; -: Feature not selected; OBJ 1: Bold indicates the selected optimal solution. OBJ 2: (Numerator count + Denominator count) in parentheses. Variable allocation is automatically optimized by the MONNF algorithm according to the following rules: Positive risk prognosticators are assigned exclusively to the numerator, negative protective indicators are restricted to the denominator, and U-shaped variables use |actual value - inflection point| in the numerator

### Prognostic predictive value analysis

#### Single-indicator analysis

1. ROC Curves

**Fig. 4:**
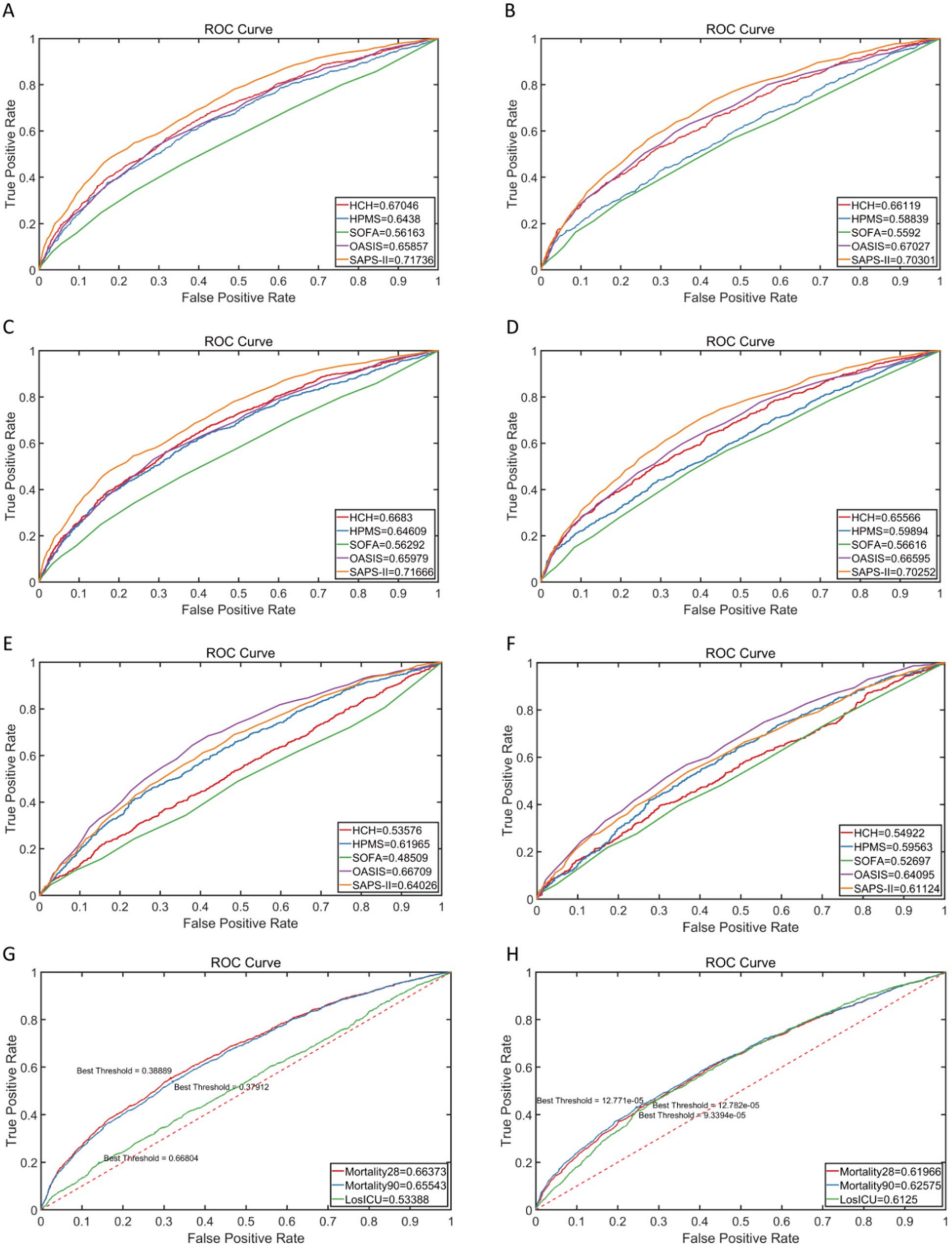
ROC curve analysis of novel indicators for predicting sepsis prognosis. Note: Panels **A**, **C**, **E** show the predictive performance of Hypoxia-Coagulation-Hemoglobin Index (HCH) for 28-day mortality (**A**), 90-day mortality (**C**), and ICU length of stay (**E**); Panels B, D, F demonstrate the discriminative ability of Hepatic-Pulmonary-Metabolic Synergistic Index (HPMS) for 28-day mortality (**B**), 90-day mortality (**D**), and ICU admission requirement (**F**); Panels **G**, **H** present the optimal clinical cutoff values for HCH (**G**) and HPMS (**H**) determined using the Youden index maximization method

ROC curve analysis of HCH and HPMS indices for predicting 28-day mortality, 90-day mortality, and ICU length of stay in septic patients (Fig. 4) demonstrated: Both HCH and HPMS showed higher area under the curve (AUC) values than conventional SOFA scores, indicating better predictive performance. Compared to OASIS scores, HCH maintained superior AUC values while HPMS showed comparable performance. Both HCH and HPMS exhibited slightly lower AUC values than SAPS-II scores.

(2) Restricted Cubic Spline Analysis

**Fig. 5:**
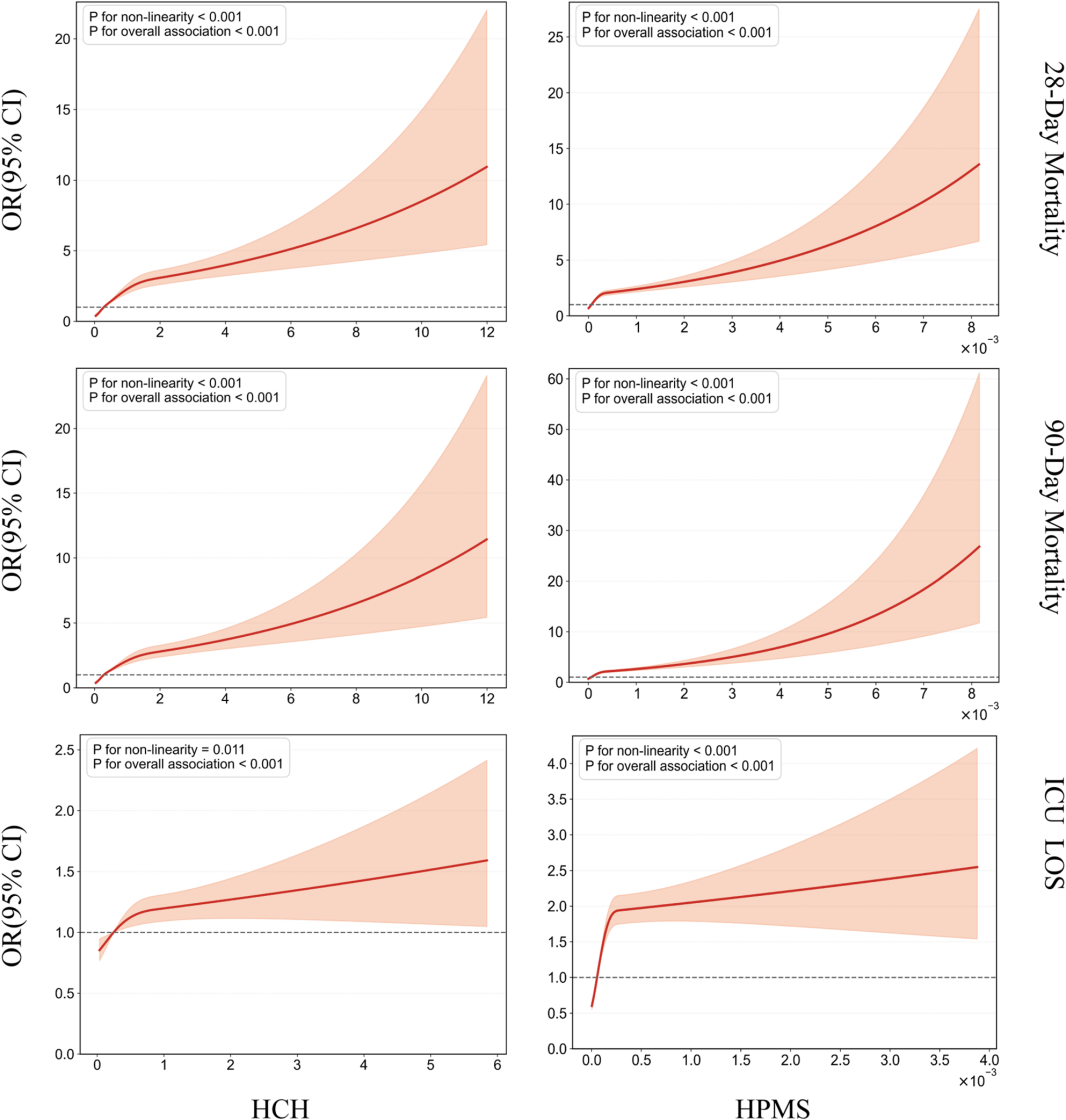
Restricted cubic spline analysis of novel indicators and sepsis prognosis outcomes. Note: The outcome measures included 28-day mortality, 90-day mortality and ICU length of stay (ICU LOS), where ICU LOS was dichotomized into whether ICU stay exceeded 7 days in 90-day survivors. The analysis used single-indicator logistic regression restricted cubic splines with 5 knots uniformly set for all models.

The restricted cubic spline analysis (Fig. 5) demonstrated a three-phase nonlinear relationship between HCH/HPMS indices and adverse sepsis outcomes. When the index values were below certain cutoff points, their association with poor prognosis was weak. However, once exceeding these thresholds, the risk level surged sharply with significantly increased curve slope. With continued elevation of the indices, the curve entered a relatively stable fluctuating phase where risk values remained high but with diminishing growth rate. At extremely high value ranges, the risk curve exhibited renewed acceleration, suggesting an amplifying effect of extreme abnormal values on prognostic deterioration.

(3) Boruta Feature Importance Ranking

**Fig. 6:**
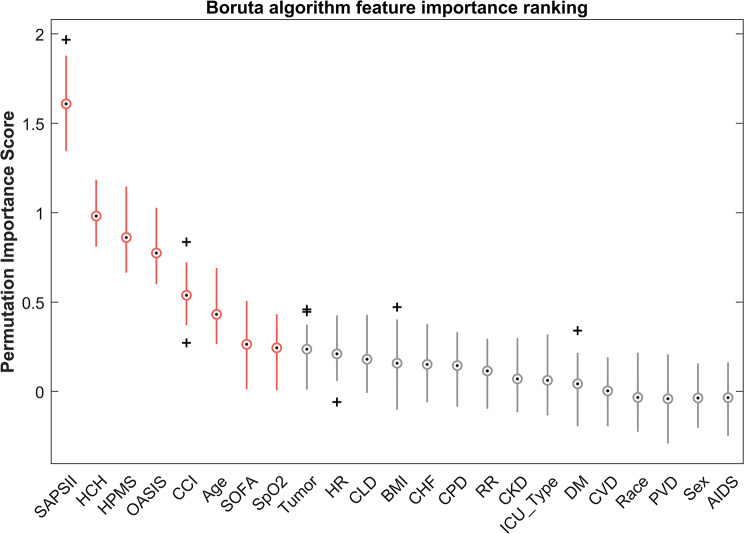
Importance ranking of key prognostic variables in sepsis patients based on Boruta algorithm. Note: All baseline data were included in the analysis. The plus signs (+) indicate outliers, and the red lines mark the selected features

Using the Boruta algorithm to rank the importance of all baseline features, the results showed HCH and HPMS ranked second and third, respectively, following only the SAPS II indicator, demonstrating high importance weights. Details are shown in Fig. 6.

#### Predictive model construction

1. Feature Selection

During model construction, to avoid information redundancy between traditional disease severity scores (including SAPS II, OASIS, and SOFA scores) and novel biomarkers, while improving the clinical translation feasibility for rapid bedside prediction, the study adopted a two-stage feature screening strategy: First, composite scoring variables with theoretical overlaps were removed, followed by selection of core predictive factors using the Boruta feature importance algorithm (number of iterations = 30).

**Fig. 7:**
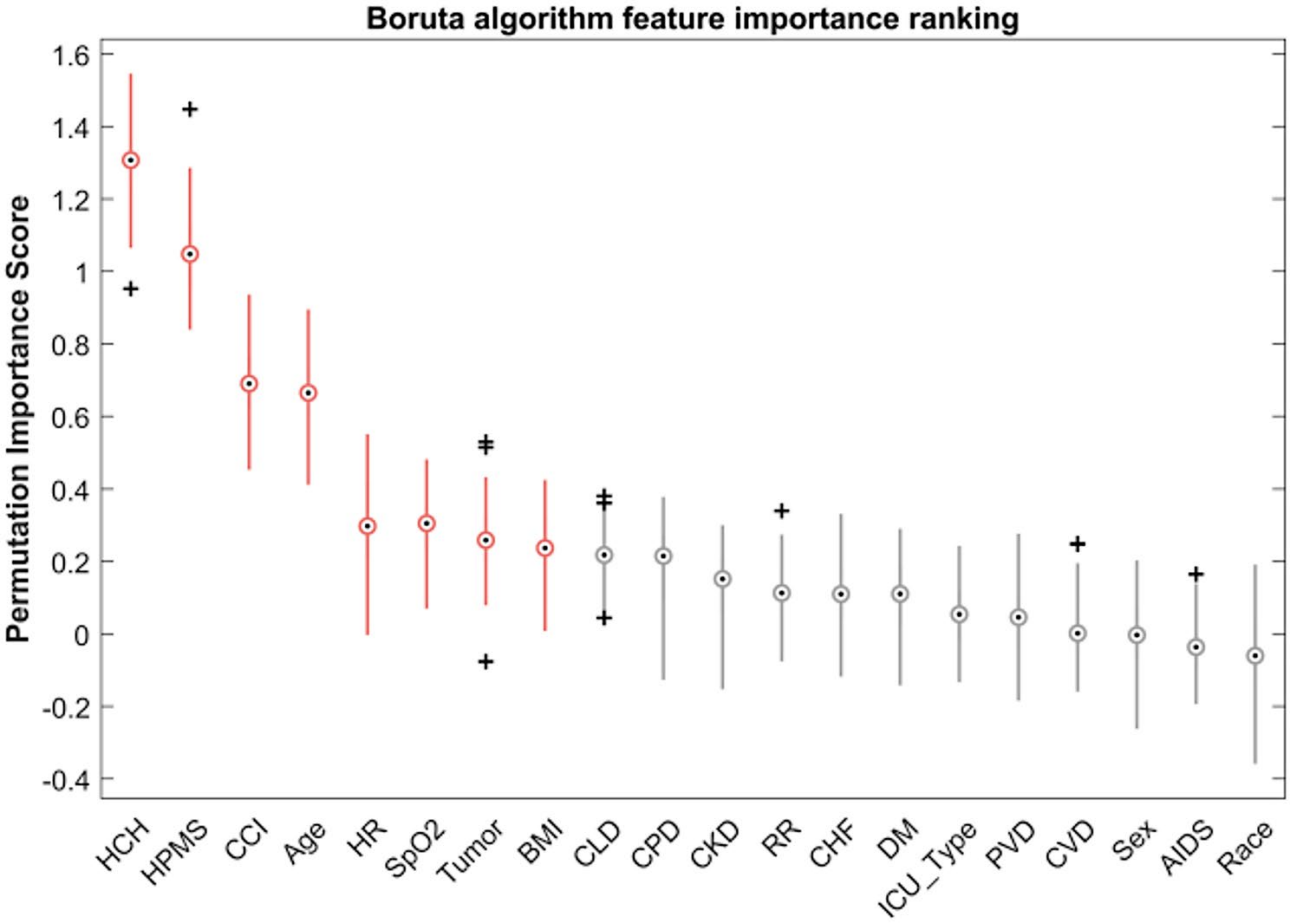
Importance ranking of key prognostic variables in sepsis patients based on Boruta algorithm. Note: Analysis was performed after removing disease severity scores (SAPS-II, SOFA, and OASIS). The plus signs (+) indicate outliers, and the red lines mark the selected features

The final established feature set, ranked in descending order of predictive contribution, was: HCH, HPMS, CCI, heart rate (HR), age (Age), oxygen saturation (SpO2), and oncologic complications (Tumor). Details are shown in Fig. 7.

(2) Model Training and Validation

**Fig. 8:**
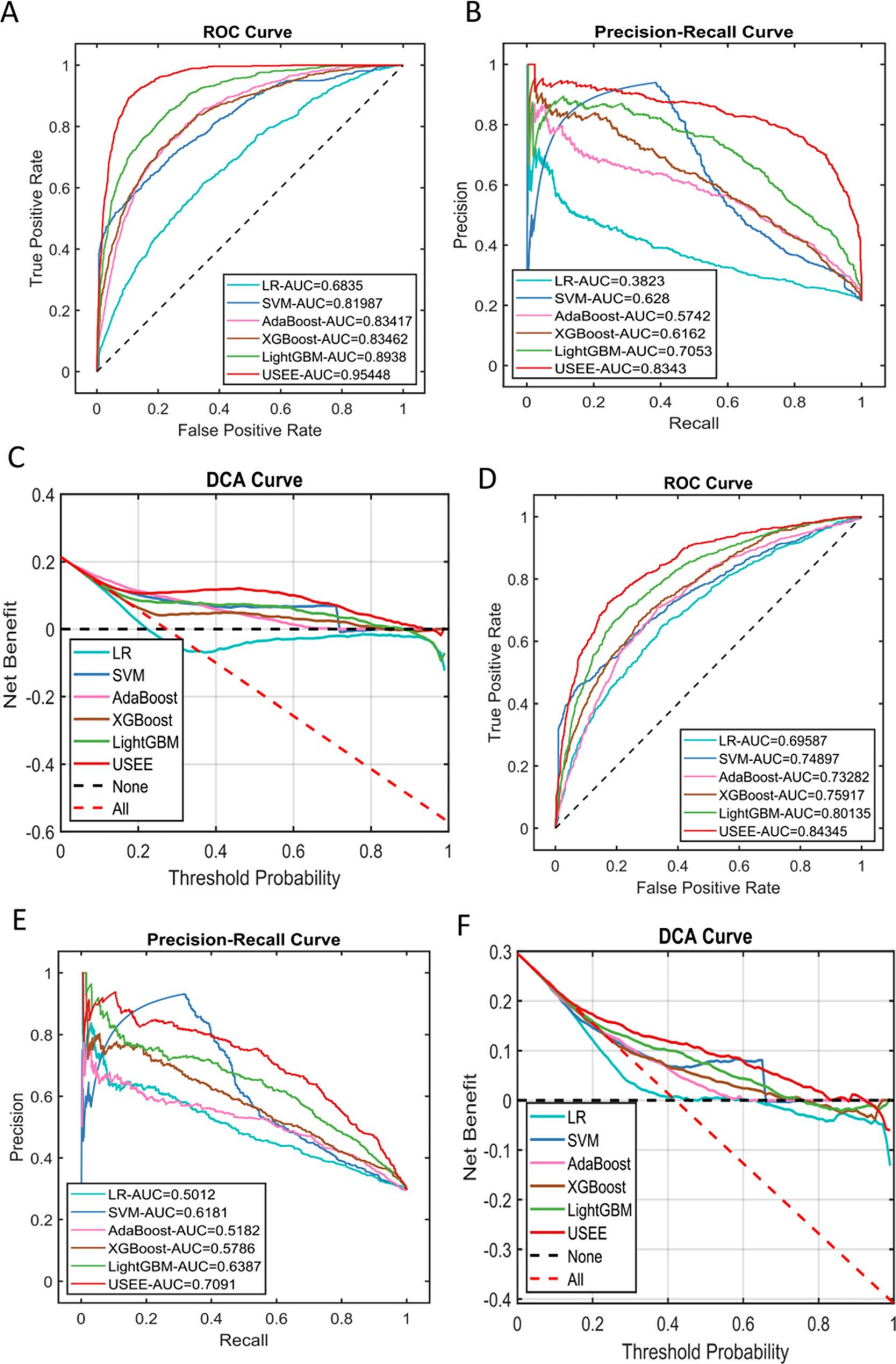
Model Training and Validation Performance. Note: Panels **A**, **B**, **C**: ROC curve, PR curve, and decision curve for the training set; Panels **D**, **E**, **F**: ROC curve, PR curve, and decision curve for the test set

**Fig. 9:**
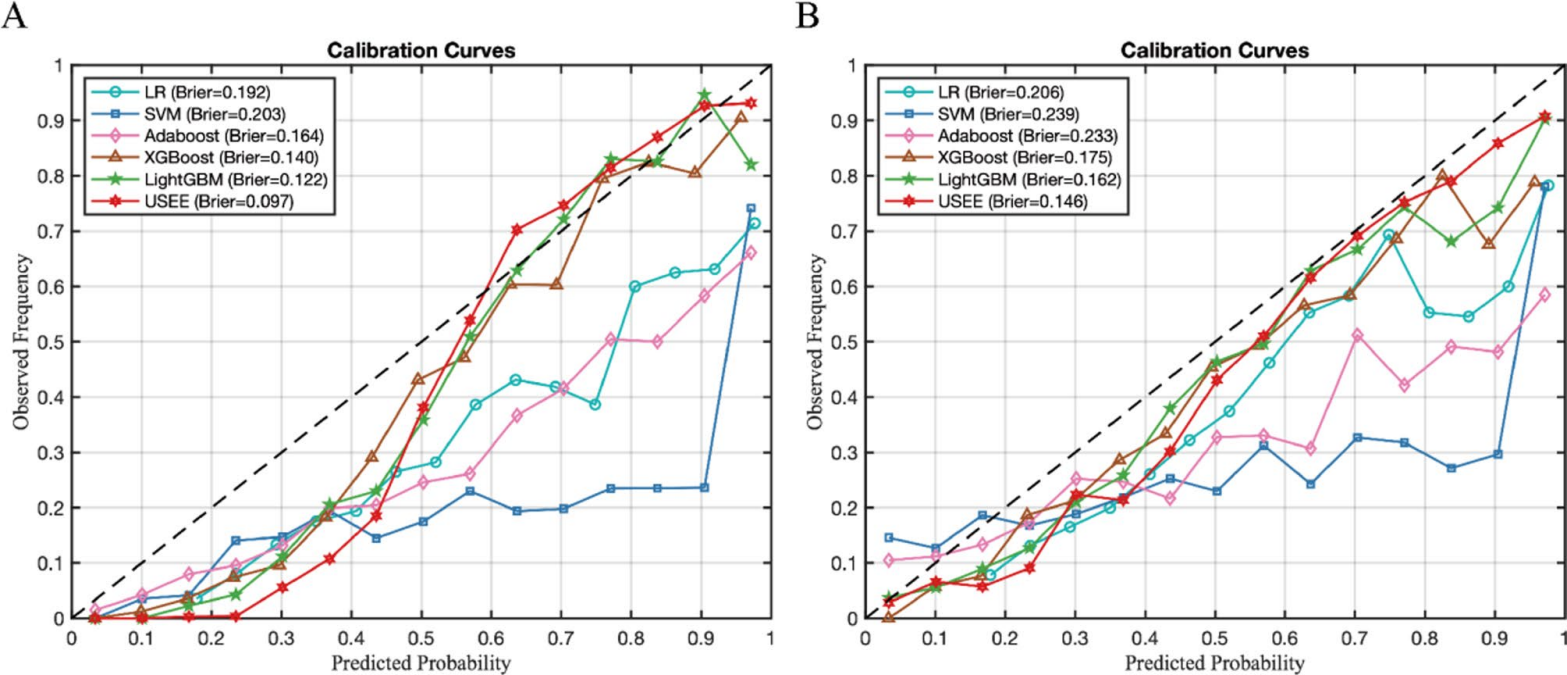
Model calibration performance. Note: Panel **A**: Calibration curve of the training set; Panel **B**: Calibration curve of the test set

The five-fold cross-validation results on the eICU-CRD v2.0 training set demonstrated that the USEE model showed significant advantages in comprehensive predictive performance compared to five baseline methods, with key performance metrics reaching F1-score = 0.67845, ROC-AUC = 0.9545, and PR-AUC = 0.8343. When validated on the independent MIMIC-IV v3.1 test set, USEE exhibited excellent generalization capability with stable metric consistency, achieving key performance metrics: F1 = 0.6584, ROC-AUC = 0.8435, and PR-AUC = 0.7091. Details are shown in Table 5; Fig. 8. The calibration curve analysis (Fig. 9) confirmed that the predictive calibration performance of the USEE model was significantly superior to other models, with both the training set Brier score (0.097) and test set Brier score (0.146) being the lowest.

**Table 5:** Model training performance and generalization validation on test set

Dataset	Model	PRE	SEN	SPE	ACC	F1	ROC-AUC	PR-AUC
Training Set	LR	0.4044	0.3663	0.8530	0.7488	0.3844	0.6835	0.3823
	SVM	0.5271	0.6078	0.8514	0.7992	0.5646	0.8198	0.6280
	Adaboost	0.4768	0.7256	0.7831	0.7707	0.5754	0.8342	0.5742
	XGBoost	0.6264	0.5135	0.9166	0.8303	0.5644	0.8346	0.6162
	LightGBM	0.6579	0.6749	0.9044	0.8552	0.6663	0.8938	0.7053
	USEE	0.7290	0.8492	0.9140	0.9001	**0.7845**	**0.9545**	**0.8343**
Test Set	LR	0.5105	0.4513	0.8185	0.7100	0.4791	0.6959	0.5012
	SVM	0.5024	0.6148	0.7445	0.7062	0.5529	0.7490	0.6181
	Adaboost	0.4740	0.6775	0.6847	0.6826	0.5578	0.7328	0.5182
	XGBoost	0.6073	0.4432	0.8798	0.7508	0.5124	0.7592	0.5786
	LightGBM	0.6448	0.5580	0.8710	0.7785	0.5983	0.8014	0.6387
	USEE	0.6699	0.6473	0.8662	0.8015	**0.6584**	**0.8435**	**0.7091**

Note: Bold numbers indicate comparatively better performance in comparative evaluation of comprehensive metrics

(3) Interpretability Analysis

**Fig. 10:**
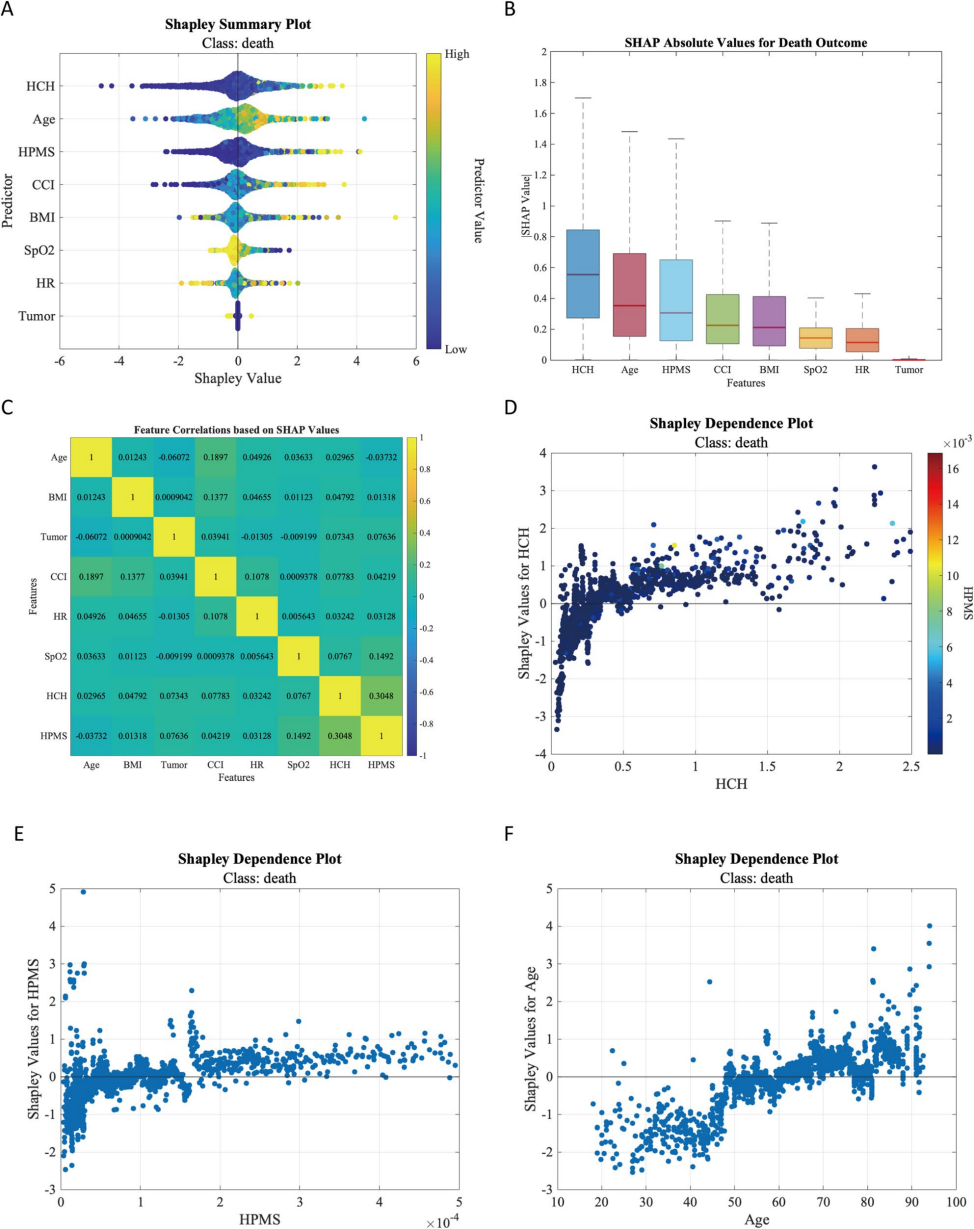
Interpretability analysis of machine learning models. Note: **A**: Shapley summary plot; **B**: Box plot of absolute Shapley values; **C**: Heatmap of Shapley value correlations across indicators; **D**: Shapley dependence plot for HCH-HPMS interaction; **E**: Shapley dependence plot for HPMS; **F**: Shapley dependence plot for Age

Global feature analysis based on the SHAP framework revealed that the feature contribution pattern of the sepsis prognosis risk prediction model exhibited a stepwise hierarchical structure. The strongest predictive factor with the strongest decision contribution was HCH, followed successively by Age, HPMS, CCI, BMI, HR, SpO₂, and Tumor complications (Fig. 10A-B).

The Shapley values among different features showed a low-correlation distribution (Fig. 10C), indicating good feature independence. Shapley dependence analysis further demonstrated that when HCH and HPMS exceeded specific cutoff values, their associations with adverse outcomes tended to reach plateaus (Fig. 10D-E), indicating the existence of threshold effects in risk assessment.

## Discussion

Sepsis, defined as a multiple organ dysfunction syndrome triggered by dysregulated host responses to infection, involves complex metabolic-immune-coagulation network imbalances. The dynamic heterogeneity of its pathological progression poses significant challenges for early precise assessment [18, 43]. While conventional scoring systems can quantify organ dysfunction severity, their reliance on multi-parameter measurements across multiple timepoints often delays golden-window interventions through complex computational requirements. Static evaluation frameworks exhibit fundamental limitations in capturing synergistic effects among dynamic indicators such as lactate fluctuations and coagulation disorders [44].

To address this clinical bottleneck, the MONNF developed in this study identifies novel composite indicators from core pathological dimensions of sepsis, offering a new approach to balance assessment efficiency with predictive performance. The MONNF framework overcomes limitations of conventional optimization algorithms through three core innovations: its physics-inspired dynamic search strategy autonomously discerns topological features of data distributions while adaptively switching between global exploration and local optimization; its multi-objective synergistic viscoelastic constraints simulate cascade effects of multi-organ injury; and embedded domain-knowledge rules encode clinical relevance into optimization logic, ensuring selected indicators demonstrate both statistical validity and medical interpretability. Compared with existing intelligent algorithms, this mechanism-driven optimization paradigm not only enhances pathological pattern representation but also pioneers new pathways for AI in critical care medicine through deep integration of biological principles with mathematical models.

The HCH indicator exemplifies the clinical value of this innovative framework. By integrating three conventional parameters (lactate, INR, and hemoglobin), this metric captures key patterns of vicious cycle mechanisms in sepsis deterioration through dual dimensions: circulatory perfusion impairment and coagulation-oxygenation dysfunction. Elevated lactate associates with metabolic crisis from tissue hypoperfusion, abnormal INR reflects endothelial injury from coagulopathy, and low hemoglobin coincides with oxygen supply-demand imbalance [18, 37]. Compared to traditional critical care scores, HCH achieves comparable predictive power with streamlined parameters, significantly reducing clinical data acquisition complexity—may demonstrate utility in emergency triage pending validation. Moreover, the tipping-point effect revealed by its dynamic risk curve establishes critical thresholds between compensatory and decompensatory phases, suggesting potential clinical decision thresholds.

Building upon HCH’s focus on circulatory-coagulatory dysfunction, the HPMS indicator further associates with multi-organ dysregulation linked to prognosis. The composite metric—comprising total bilirubin, oxygenation index, bicarbonate, and albumin—maps core pathological processes: hepatobiliary metabolic failure (bilirubin), impaired pulmonary oxygenation (PaO₂/FiO₂), metabolic acidosis (bicarbonate), and nutritional exhaustion (albumin), constructing an early-warning model from hepatopulmonary-metabolic interplay [30, 45, 46]. Its discriminative power significantly surpasses single-organ scores, validating the advantage of multi-system modeling for sepsis complexity. The threshold-triggered exacerbation pattern observed in its nonlinear risk curve not only aligns with clinical progression of multiple organ failure but also suggests potential quantitative allocation criteria for critical care resources.

For potential clinical implementation, elevated levels of the optimized indices could prompt intensified monitoring protocols: HCH threshold exceedance may warrant enhanced surveillance of perfusion-coagulation-hematologic axis, HPMS elevation could indicate need for hepatopulmonary-metabolic function reassessment. Such alert-response pathways would require prospective validation to determine: (1) Optimal monitoring frequency upon index deviation; (2) Most effective interventions for index-specific abnormalities; (3) Impact on patient-centered outcomes in goal-directed therapy.

It must be emphasized that the USEE prediction model employed in this study effectively addresses the intertwined challenges of class imbalance and overfitting through optimized sample processing strategies and algorithmic architecture [38]. Cross-center data validation demonstrates its generalization performance significantly surpasses traditional machine learning models, while interpretability analysis indicates that novel indicators show dominant decision contributions with association patterns highly consistent with clinical cognition. This interpretable model characteristic allows clinicians to understand prediction patterns. Furthermore, while SHAP values provide mathematical attribution of feature contributions, their biological relevance requires validation through dedicated mechanistic studies. Future work should experimentally verify whether the hierarchical patterns observed in SHAP analysis (e.g., HCH as primary predictor) correspond to actual causal pathways in sepsis pathogenesis.

Our approach, combining the MONNF algorithm for indicator discovery with the USEE predictive model, presents distinct advantages when compared to several state-of-the-art sepsis predictive models [47–51]. While many existing machine learning models focus on improving predictive accuracy using a black-box approach, often relying on a large number of heterogeneous features, our framework prioritizes mechanistic interpretability and clinical feasibility from the outset. Specifically, the MONNF algorithm is designed not merely for predictive power but to uncover concise, pathologically synchronized composite indicators (HCH and HPMS). Unlike models that may directly incorporate hundreds of raw variables, leading to complex and less interpretable outputs [47, 49], our approach reduces dimensionality by constructing physiologically meaningful indices. This allows clinicians to easily grasp the underlying pathological essence (e.g., circulatory compromise, hepatopulmonary dysfunction) represented by the HCH and HPMS, fostering better clinical understanding and potentially actionable insights, which is often a challenge for highly complex ML models [51]. For instance, a recent study leveraging routine complete blood count parameters with machine learning for sepsis detection represents an important step towards simplifying input features [48]. Our work extends this by not only selecting routine parameters but synthesizing them into novel, interpretable composite indices that explicitly map to distinct pathological domains.

Furthermore, our MONNF’s unique physics-inspired dynamic search strategy and domain-knowledge embedding ensure that the derived indicators are clinically rational, avoiding combinations that are statistically robust but pathophysiologically incongruent – a common pitfall in purely data-driven feature selection [50]. The ability of our USEE model to achieve an optimal predictive performance on independent validation, while simultaneously emphasizing the dominant decision contributions of these interpretable novel indicators, highlights a successful integration of high accuracy with strong interpretability. This contrasts with many contemporary models where interpretability remains a post-hoc analysis rather than an inherent design principle. Our framework’s explicit generation of indices like HCH and HPMS offers a simplified yet powerful tool for clinical stratification, directly addressing the “tripartite paradox” of efficiency, sensitivity, and interpretability in sepsis assessment.

This study has certain limitations. First, the screening of novel indicators relies on existing laboratory parameter systems, and the exclusion of emerging biomarkers may restrict their analytical capacity for mechanisms such as immune dysregulation. Second, models derived from retrospective data require validation through prospective cohorts to establish their temporal stability. Additionally, the clinical utility of novel indicators necessitates interventional studies to determine their impact on improving patients’ final outcomes. Future research could explore integration with technologies such as microcirculation monitoring and cytokine profiling to construct a more comprehensive early-warning ecosystem for sepsis. Furthermore, while we have adhered to the TRIPOD + AI guideline to enhance transparency, the retrospective design based on administrative databases may include unmeasured confounding factors and is subject to the limitations of data quality and coding practices inherent to such sources. The clinical utility of the HCH and HPMS indices must be prospectively validated in interventional settings.

## Conclusion

This study developed a multi-objective optimization algorithm (MONNF) to extract multidimensional features from sepsis patients in the MIMIC-IV/eICU-CRD databases, culminating in two novel composite prognostic indicators—HCH and HPMS. A triple-validation framework integrating ROC curves, restricted cubic splines, and Boruta algorithms confirmed their individual discriminative power. An evolutionary ensemble learning model was subsequently constructed. Interpretability analysis (SHAP) visualized stepwise hierarchical contribution pattern of feature contributions but also identified a risk-interaction threshold effect between HCH and HPMS. These findings present methodological innovations to potentially optimize clinical stratification decisions and represent preliminary steps toward balancing efficiency, sensitivity and interpretability involving efficiency, sensitivity, and interpretability in prognostic assessment.

## Supplementary Information

Below is the link to the electronic supplementary material.

Supplementary Material 1

Supplementary Material 2

## Data Availability

This study was performed with the data from the Medical Information Mart for Intensive Care (MIMIC)-IV version 3.1 and the eICU Collaborative Research Database (eICU-CRD) version 2.0. Even though datasets are de-identified, restrictions have been imposed on data sharing since they contain sensitive information. Conventions are signed for researchers before any access to the data. For data access, interested researchers must fulfill all of the following requirements: be a credentialed user of https://physionet.org/, finish required training and sign the data use agreement for the project.
