# Supplementary material for: Development and external validation of the HCH and HPMS prognostic indices for sepsis: a retrospective model development study using a Multi-Objective Non-Newtonian Fluid optimization algorithm
Source: BMC Med Inform Decis Mak. 2026 Jun 2;26:298. doi: 10.1186/s12911-026-03609-8 (PMC13449319; doi:10.1186/s12911-026-03609-8)
Supplement: Supplementary file 1 [file 12911_2026_3609_MOESM2_ESM.pdf]

Supplementary File 2:

Design Principles and Validation of the Non-Newtonian Fluid Optimization Algorithm

Traditional swarm intelligence algorithms (e.g., Particle Swarm Optimization, Ant Colony Optimization) predominantly draw inspiration from Newtonian mechanics, assuming linear relationships between interaction forces and velocities among individuals. However, real-world optimization problems often exhibit nonlinearities in the search space and multimodal local optima distributions, demanding algorithms with dynamic adaptability and multimodal exploration capabilities.

Inspired by the complex rheological properties of non-Newtonian fluids, this paper proposes a novel Non-Newtonian Fluid Optimization algorithm (NNF). By mimicking characteristic non-Newtonian phenomena—including shear thinning, jet swelling, and tubeless siphoning—the algorithm achieves:

(1) Dynamic perception of gradient information in the solution space

(2) Self-adaptive exploration strategies

(3) Effective equilibrium between global exploration and local exploitation

The core innovation lies in translating fluidic viscoelastic behaviors into mathematical operators that autonomously respond to topological features of the fitness landscape, thus overcoming limitations of conventional physics-inspired approaches.

1.Physically-Inspired Optimization Mechanics

1.1 Core Principles

Non-Newtonian fluids exhibit stress-strain relationships that dynamically depend on shear rates—an ideal metaphor for adaptive optimization:

(1) Viscosity (η) ➔ Adaptive weights: Govern movement magnitude

(2) Shear thinning ➔ Accelerated convergence

(3) Rod climbing effect ➔ Reverse exploration

(4) Tubeless siphoning ➔ Cross-region search

(5)Turbulent drag reduction ➔ Perturbation injection

1.2 NNF Pseudocode

```
pop = rand(popSize,dim);              % Init
G = top3(pop);                        % Guides [G1,G2,G3]
S = zeros(popSize,1);                 % Stag counters

for t = 1:Tmax
    s = 1;
    if ~categoryCoverage(pop,G) % Jet swell check
        s = 2*rand()-1;               % Expansion [-1,1]
    end

    for i = 1:popSize
        % Main update strategies
        if S(i) > 0.1*Tmax                        % Stagnation escape
            X_new = randn*G(randi(3),:);
        elseif pop(i) == any(G)                   % Guide update
            X_new = G(1,:) + s*rand*vec(G1,G2);
        else                                      % Fluid dynamics
            k = randi(4);                         % Model selector
            X_new = move(i,G(k,:),s,t/Tmax,k);
        end

        % Update logic
        if f(X_new) < f(pop(i,:))
            pop(i,:) = X_new;
            S(i) = 0;                 % Reset counter
        else
            S(i) = S(i)+1;            % Increase counter
        end
    end

    G = top3(pop);                    % Update guides
    conv(t) = f(G(1,:));              % Track best
end
best = G(1,:);
```

2. Algorithmic Formulation of NNF

2.1 Notation

（1） $X_i^t = [x_{i1}^t, x_{i2}^t, \ldots, x_{id}^t]$： Position of agent *i* at iteration *t*

（2） $f(X)$： Objective fitness function

（3） $G^t = \{g_1^t, g_2^t, g_3^t\}$： Guide points (top-3 solutions)

（4） $c_i^t \in \{1,2,3\}$： Cluster label of agent i

2.2 Guide Points and Dynamic Clustering

Step 1: Select guide points:

$$g_k^t = \arg\min_{X \in P^t} f(X), \quad k = 1,2,3 \tag{0.1}$$

where is P^t the population at t-th iteration.

Step 2: Assign clusters via Euclidean distance:

$$c_t^t = \arg\min_k \left\| X_i^t - g_k^t \right\|_2 \tag{0.2}$$

Tubeless Siphoning Enhancement: $c_{g_2}^t \leftarrow 1$, $c_{g_3}^t \leftarrow 1$ to accelerate convergence.

2.3 Jet Swelling Effect

If $\exists k \notin \{c_i^t\}$, activate random expansion:

$$swollen^t = \begin{cases} 2 \cdot U(0,1) - 1, & \text{empty cluster exists} \\ 1, & \text{otherwise} \end{cases} \tag{0.3}$$

Simulates diameter expansion when fluid exits capillaries.

2.4 Non-Newtonian Update Strategies

The position update rule is based on the physical properties of different non Newtonian fluids, as shown in Table S1.

Table S1 Selecting four typical non Newtonian behaviors based on probability

Fluid Type	Probability	Physical Analogy	Optimization Role
Shear-thickening	25%	$\eta\uparrow$ with shear rate	Late-stage refinement
Shear-thinning	25%	$\eta\downarrow$ with shear rate	Early-stage exploration
Bingham plastic	25%	Yield stress (τ>0)	Mid-iterative activation
Viscoelastic	25%	Normal stress reversal	Reverse-gradient escape

Position update:

$$X_i^{t+1} = swollen^t \cdot X_i^t + \Delta X_i^t + \zeta \tag{0.4}$$

Where $\zeta \sim U(0,10^{-5})$ avoids stagnation.

Displacement vectors:

（1）Shear-thickening (Suppresses oscillation):

$$\Delta X_i^t = r_1 \cdot (1-(\frac{t}{T})^2) \cdot (g_{c_i}^t - X_i^t) \tag{0.5}$$

（2）Shear-thinning (Boosts exploration):

$$\Delta X_i^t = r_2 \cdot (1-(\frac{T-t}{T})^2) \cdot (g_{c_i}^t - X_i^t) \tag{0.6}$$

（3）Bingham plastic (Delayed activation):

$$\Delta X_i^t = \begin{cases} 0, & t \le T/2 \\ r_3 \cdot (g_{c_i}^t - X_i^t), & t > T/2 \end{cases} \tag{0.7}$$

（4）Viscoelastic (Reverse exploration):

$$\Delta X_i^t = -r_4 \cdot (g_{c_i}^t - X_i^t) \tag{0.8}$$

Where $r_1 \sim r_2 \sim U(0,1)$ is the random coefficient and T is the total number of iterations.

2.5 Turbulent Drag Reduction

If fitness stagnates for $\lfloor 0.1T \rfloor$ iterations:

$$X_i^{t+1} = \text{swollen}^t \cdot X_{\text{rand}} + \sum_{k=1}^{3} \omega_k (g_k^t - X_{\text{rand}}) \tag{0.9}$$

Where $\omega = [0.5, 1/3, 1/6]$ is the decay weight and X_{rand} is the uniform random solution.

2.6 Boundary Constraints

Project infeasible solutions back to domain:

$$x_{ij}^{t+1} = \begin{cases} lb_j, & x_{ij}^{t+1} < lb_j \\ ub_j, & x_{ij}^{t+1} > ub_j \\ x_{ij}^{t+1}, & else \end{cases} \tag{0.10}$$

3. Extension to Multi-Objective Optimization (MONNF)

The NNF algorithm is extended to multi-objective optimization through an archive mechanism that preserves non-dominated solutions, enabling dynamic tracking of the Pareto frontier. Non-dominated sorting is implemented as follows:

(1) **Non-dominated Sorting:** Evaluate the population to compute the domination count n_i (number of solutions dominating individual i) and dominated solution set S_i (solutions dominated by individual i).

(2) **First Front Construction:** Assign all individuals with n_i=0 to the first Pareto front.

(3) **Iterative Front Generation:** Update domination counts of remaining solutions, then iteratively: Assign solutions with updated n_i=0 to the next front; Repeat until all individuals are sorted into successive frontiers.

4. Algorithm Performance Simulation Testing

To systematically evaluate the superior performance of NNF, this study selects representative traditional swarm intelligence algorithms (Genetic Algorithm GA, Particle Swarm Optimization PSO), novel intelligent optimizers (Harris Hawk Optimization HHO, Grey Wolf Optimizer GWO, Egret Swarm Optimization Algorithm ESOA), and the GA-PSO hybrid model based on a collaborative strategy as benchmark references. The validation scenarios are constructed using 12 typical functions from the CEC2022 standard test set (including unimodal, multimodal, and composite forms). The experiments strictly adhere to standardized evaluation procedures, with key parameters set as follows: solution space dimension (D=10), population size (N=30), and maximum evolution iteration count (T=500).

The boxplot visualization analysis of the global solution set distribution (Fig. S1) demonstrates that NNF exhibits significant convergence accuracy advantages in 91.67% of the benchmark scenarios (11/12 functions). The convergence curve evolution trajectory analysis (Fig. S2) further reveals that the framework demonstrates more efficient gradient descent characteristics during the iterative optimization phase.

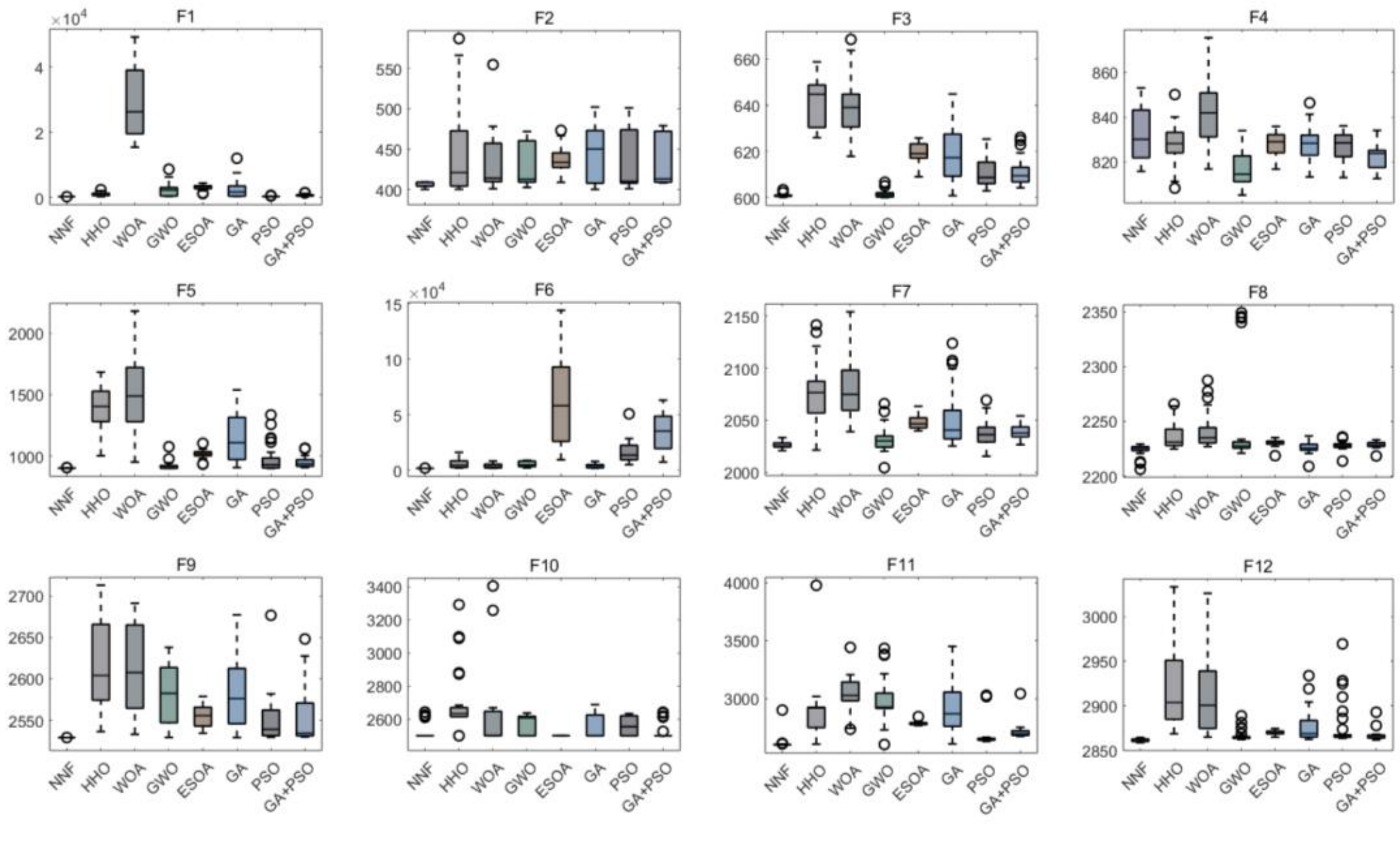

Fig. S1 Comparison of Optimization Performance

Note: The boxplots display the optimization results of each algorithm after 30 independent runs on the CEC2022 test functions, demonstrating their optimization stability and robustness.

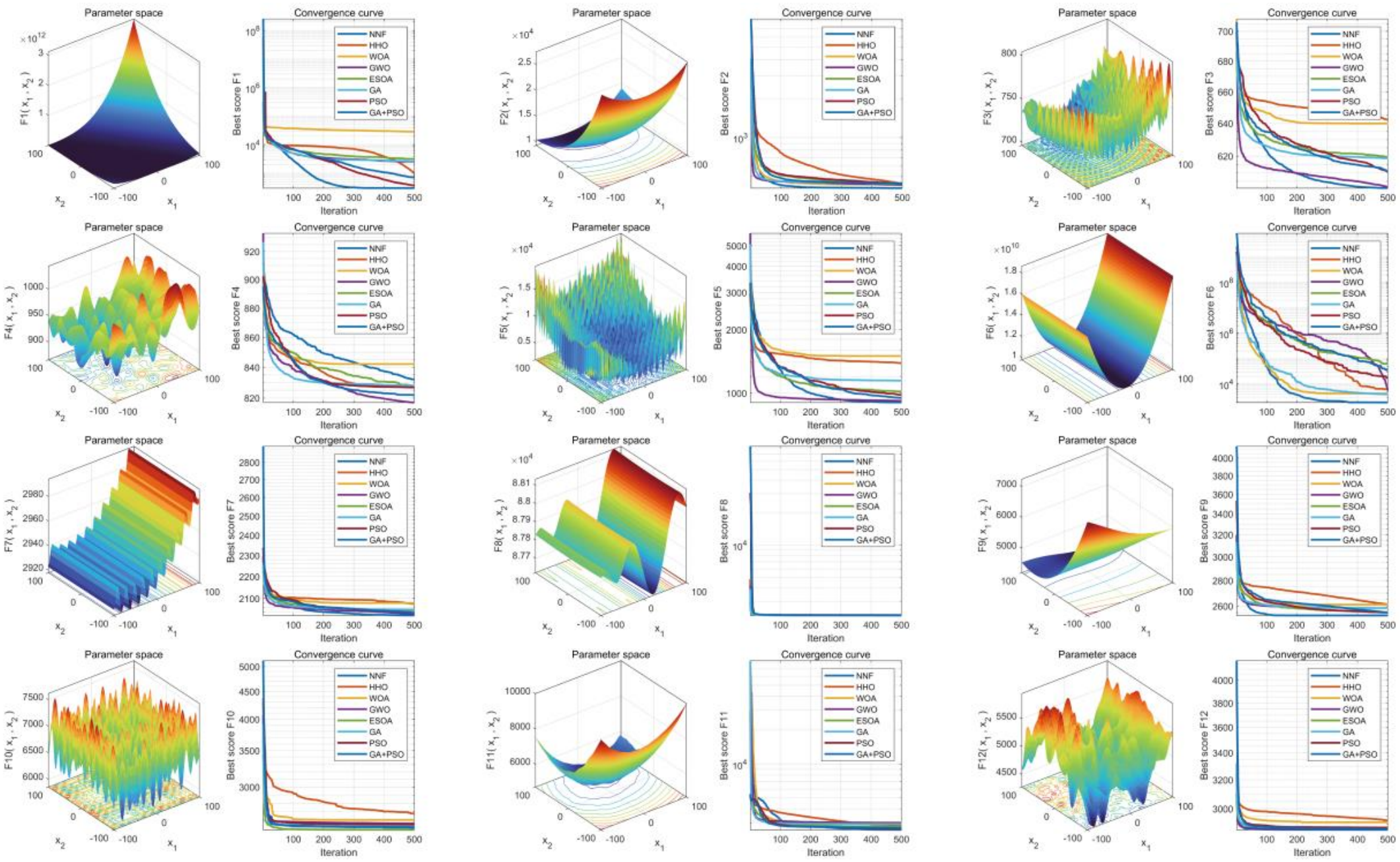

Fig. S2 Comparison of Convergence Performance

Note: The convergence curves of each algorithm during optimization reflect their convergence speed and ability to avoid local optima.
